# Hepatic Macrophages as Targets for the MSC-Based Cell Therapy in Non-Alcoholic Steatohepatitis

**DOI:** 10.3390/biomedicines11113056

**Published:** 2023-11-14

**Authors:** Irina V. Kholodenko, Konstantin N. Yarygin

**Affiliations:** Laboratory of Cell Biology, Orekhovich Institute of Biomedical Chemistry, 119121 Moscow, Russia; kyarygin@yandex.ru

**Keywords:** NAFLD, NASH, Kupffer cells, macrophages, cell death, DAMPs, mesenchymal stem cells

## Abstract

Non-alcoholic steatohepatitis (NASH) is a serious public health issue associated with the obesity pandemic. Obesity is the main risk factor for the non-alcoholic fatty liver disease (NAFLD), which progresses to NASH and then to end-stage liver disease. Currently, there are no specific pharmacotherapies of NAFLD/NASH approved by the FDA or other national regulatory bodies and the treatment includes lifestyle adjustment and medicines for improving lipid metabolism, enhancing sensitivity to insulin, balancing oxidation, and counteracting fibrosis. Accordingly, further basic research and development of new therapeutic approaches are greatly needed. Mesenchymal stem cells (MSCs) and MSC-derived extracellular vesicles prevent induced hepatocyte death in vitro and attenuate NASH symptoms in animal models of the disease. They interact with hepatocytes directly, but also target other liver cells, including Kupffer cells and macrophages recruited from the blood flow. This review provides an update on the pathogenesis of NAFLD/NASH and the key role of macrophages in the development of the disease. We examine in detail the mechanisms of the cross-talk between the MSCs and the macrophages, which are likely to be among the key targets of MSCs and their derivatives in the course of NAFLD/NASH cell therapy.

## 1. Introduction

Obesity is a serious public health problem. It is associated with multiple comorbidities, including type 2 diabetes, cardiovascular diseases, and cancers [1]. Obesity is also one of the main and most common risk factors for the non-alcoholic fatty liver disease (NAFLD). Progressive NAFLD, in 60% of cases, leads to non-alcoholic steatohepatitis (NASH); 41% of patients with NASH develop liver fibrosis, which progresses further to cirrhosis in 22% of patients, of which 2% end up with hepatocellular carcinoma within 3 years [2]. In NASH, hepatocytes undergo steatosis, while ongoing inflammation results in tissue fibrosis and end-stage liver disease [3,4]. Although the rate and course of the NAFLD/NASH progression varies among individuals, it typically proceeds through four stages: (1) lipid accumulation in the liver (NAFLD), (2) early NASH, which, in addition to fat accumulation, is also characterized by liver tissue inflammation, (3) fibrosis, manifested by chronic liver inflammation, tissue damage, and excessive accumulation of extracellular matrix (ECM) proteins, and (4) cirrhosis, the most dangerous stage of NASH, which evolves into generally fatal (without organ transplantation) end-stage liver disease [5].

More than 20 years ago, Day and Saksena [6] proposed the ‘two-hit’ theory of NASH pathogenesis. According to this theory, the first hit is steatosis, which sensitizes the liver to the second hit, rendered by some of a plethora of factors including oxidative stress, endotoxins, lipotoxicity, and necroinflammation. The accumulation of lipids such as diacylglycerol increases insulin resistance, mitochondrial and endoplasmic reticulum stress, and autophagic defects, collectively known as lipotoxicity. This event triggers immune responses in Kupffer cells and hepatic stellate cells, promotes tissue infiltration with neutrophils and lymphocytes, and ultimately leads to fibrosis and death of hepatocytes [7]. Somewhat later, Tilg and Moschen proposed the “multiple parallel hits” hypothesis for the pathogenesis of NASH [8]. They stated that NASH is a pathogenetically complex condition that involves cross-talk between several metabolically active tissues (adipose tissue and the liver/gut axis). According to the “multiple hits” theory, multiple disorders, including insulin resistance, hepatic lipid accumulation, oxidative stress, mitochondrial dysfunction, abnormal gut microbiota characteristics, nutritional factors, and genetic and epigenetic factors, may occur in parallel [8].

Although several drugs are at the late stage of clinical development, NASH therapy is still based on lifestyle adjustment (diet, exercise, etc.) and the prescription of medicines improving lipid metabolism, enhancing sensitivity to insulin, balancing oxidation, and counteracting fibrosis. There are currently no FDA-approved pharmacotherapies for the treatment of NAFLD/NASH [9]. Therefore, new therapeutic options for NASH are greatly needed. Promising results have been obtained in a variety of preclinical studies conducted in various animal models of NAFLD/NASH using mesenchymal stem/stromal cells (MSCs) and their derivatives, including conditioned media, extracellular vesicles, and apoptotic bodies [10,11,12]. The beneficial effects of the cell therapy of many diseases with MSCs are at least partly associated with immunomodulation via direct and indirect interactions with different immune cells [13], including macrophages [14]. The reactions of Kupffer cells, the liver macrophages, to MSC transplantation have also been reported [15].

In this review, we provide an update on the pathogenesis of NAFLD/NASH and the key role of Kupffer cells and recruited macrophages in the development of inflammation and disease progression. We examine in detail the mechanisms of the cross-talk between MSCs and macrophages, which likely mediate the therapeutic effects of MSCs and their derivatives in the treatment of NAFLD/NASH.

## 2. NAFLD/NASH Pathogenesis

Inflammatory liver diseases, in the absence of contagious pathogens, are denoted as “sterile”. These conditions include xenobiotic intoxication, e.g., as a result of para-acetaminophenol (APAP) administration, cholestatic liver injury, liver ischemia–reperfusion injury (IRI), non-alcoholic steatohepatitis (NASH), and alcoholic liver disease (ALD). Triggers of pathological events in sterile liver diseases include various xenobiotics or endogenous bioactive molecules that initiate the release of intracellular compounds from living or dying parenchymal cells. It has been shown that both acute and chronic liver diseases involve the massive death of hepatocytes [16]. Extensive hepatocyte death can be and commonly is diagnosed by observing an increase in the serum levels of alanine aminotransferase (ALT) and aspartate aminotransferase (AST). These parameters have significant prognostic value for patients with NASH [17,18] and other liver diseases since they are highly correlated with overall and liver-specific mortality in the general population [19,20], highlighting the role of cell death as a major driver of liver disease progression and the development of liver fibrosis, cirrhosis, and hepatocellular carcinoma.

In liver pathologies cells die in different ways. For example, a wide range of cell death types, including apoptotic [21], necrotic [22], autophagic [23], pyroptotic [24], and ferroptotic [25] cell death were found in alcoholic liver disease (ALD). Autophagy and apoptosis are considered to be the predominant modes of hepatocyte death in ALD, while the contribution of necroptotic cell death is still a controversial issue [26]. In APAP hepatotoxicity, mitochondrial permeability transition-mediated regulated necrosis is the main type of cell death, playing the key role in liver damage [27], while the contribution of autophagy and apoptosis seems unlikely [26].

NASH is characterized by steatosis, ballooned hepatocytes containing hyaline inclusions (Mallory bodies), and severe inflammation leading to fibrosis, cirrhosis, and hepatocellular carcinoma. There is strong evidence that hepatocyte cell death is a driver of inflammation and fibrosis in NASH [28]. However, the predominant mode of cell death in this metabolic disease is still debated. Liver biopsies of patients with NASH showed increased TUNEL assay positivity and caspase-3/7 expression, indicating the prevalence of apoptotic cell death of hepatocytes in NASH [29]. Also, in a high-fat diet (HFD) mouse model of NASH, it was shown that use of the pan-caspase inhibitor Emricasan significantly attenuated liver injury, inflammation, fibrosis, and apoptosis, suggesting a critical role for apoptosis in the pathogenesis of NASH [30]. However, despite the clearly beneficial effects of the pan-caspase inhibitor in animal models and early clinical trials in a small group of NASH patients [31,32], randomized clinical trials of the pan-caspase inhibitor Emricasan conducted in a larger group of NASH patients did not show significant clinical improvement [33]. In the short term, treatment with Emricasan reduced serum ALT levels, but it not only did not improve the liver histology in patients with NASH, but even worsened fibrosis and hepatocyte ballooning. The authors of the study suggested that treatment with the pan-caspase inhibitor could provoke the onset of the alternative mechanisms of cell death inciting more pronounced fibrosis and hepatocyte ballooning [33]. Nevertheless, many studies suggest that in NASH apoptosis appears to be the predominant mode of cell death and can occur through both intrinsic (via lipotoxicity and organelle stress) and extrinsic (via cell surface receptors) pathways. Many authors do not exclude the contribution of pyroptosis and ferroptosis in the development of NAFLD/NASH. While the participation of pyroptosis in the pathogenesis of NAFLD/NASH was mainly demonstrated in animal models [34,35] and less reliably in patients with NASH [36], the hallmarks of ferroptosis, such as lipid peroxidation, ROS accumulation, and increased liver iron stores (both in the parenchymal and non-parenchymal compartment) were identified in most NASH patients [37]. In addition to apoptotic cell death of hepatocytes in NASH livers, there is also evidence of necrotic cell death. Free cholesterol accumulation in NASH patients [38,39] was demonstrated to cause apoptotic and necrotic hepatocyte death through activation of the c-Jun N-terminal kinase 1 (JNK1) [40].

Dying hepatocytes release a large number of different molecules capable of causing infection-independent activation of immune responses and their repertoire depends on the type of cell death [41]. According to Matzinger’s “danger theory” of innate immunity [41,42,43], these cell death type-specific libraries of endogenous molecules, released in all tissues in response to the non-contagious harmful stimuli and named damage-associated molecular patterns (DAMPs), are scanned by the immune cells and recognized as “self-safety” or “self-danger” signals. Because DAMPs induce inflammatory responses in the absence of pathogenic infection, the inflammation they induce is called sterile inflammation [44]. DAMPs can activate not only innate immune cells such as macrophages, neutrophils, and dendritic cells, but also non-immune cells including fibroblasts [45], hepatic stellate cells [46], and endothelial cells [47]. Activation of both immune and non-immune cells by DAMPs results in the production of various cytokines and chemokines that trigger adaptive immune responses. Although sterile inflammation plays a critical role in tissue repair, regeneration, and homeostasis, unresolved chronic inflammation leads to the development of sterile inflammatory diseases, including the liver diseases mentioned above. DAMPs comprise nuclear proteins (e.g., high mobility group box-1, HMGB-1), cytosolic proteins (e.g., keratin-18), and mitochondrial components or mitochondrial DAMPs (mtDNA), including N-formyl peptides, cardiolipin, adenosine triphosphate (ATP), mitochondrial transcription factor A (TFAM), and nucleic acids in various conformations (e.g., single-/double-stranded RNA or DNA) [48]. DAMPs can initiate immune responses through the activation of classical pattern recognition receptors (PRRs), which include membrane-bound Toll-like receptors (TLRs) and C-type lectin receptors (CLRs), cytoplasmic NOD-like receptors (NLRs), retinoic acid inducible gene I (RIG-I)-like receptors (RLRs), and multiple intracellular DNA sensors, as well as via non-PRR transmembrane proteins, including receptors for advanced glycation end products (RAGE), triggering receptors expressed on myeloid cells (TREMs), G-protein-coupled receptors (GPCRs), and ion channels [49].

NASH is a chronic state of sterile inflammation initiated by the presence of DAMPs released from damaged hepatocytes. DAMPs triggering NASH-associated inflammation have been reliably identified and involve extracellular ATP, keratin 18, HMGB1, various mtDAMPs, etc. Free fatty acid-induced lipoapoptosis in human hepatocytes stimulates ATP release, promoting monocyte recruitment [50,51]. In addition, free ATP attracts circulating neutrophils, ensuring their adhesion within the hepatic sinusoids, while mitochondrial DAMP N-formyl peptides direct neutrophils to the site of injury [52]. Lipotoxic damage also includes mitochondrial permeability transition with cytochrome c release, mitochondrial impairment, and oxidative stress. Liver biopsies from patients with NAFLD or NASH have been found to exhibit both structural and functional mitochondrial abnormalities [53]. Structural abnormalities include organelle enlargement, while functional abnormalities result in increased ROS production or accumulation of lipid peroxides [53]. Furthermore, plasma from NASH patients has recently been shown to contain intact mitochondria and high levels of oxidized mitochondrial DNA enclosed in hepatocyte-derived microparticles [54,55]. Keratin 18 is the major epithelial-specific liver intermediate filament protein expressed by hepatocytes and cholangiocytes [56,57]. In apoptosis, keratin 18 is one of the most important substrates of activated caspases generating a neo-epitope after cleavage. Caspase-cleaved fragments of keratin 18 are released into the blood as part of inclusion bodies [58,59]. During necrotic cell death, intact keratin 18 is released into the blood, where it circulates as part of extracellular vesicles [60]. Thus, the levels of fragmented keratin 18 and intact keratin 18 reflect apoptotic and total cell death, respectively, while the ratio serves as an apoptotic index [60]. Patients with NASH show an increased number of caspase-cleaved keratin 18 fragments compared to patients with simple steatosis [29]. However, the clinical assessment of the diagnostic value of the measurement of the keratin 18 fragment levels is still controversial (reviewed in [60]). Keratin 18 has so far attracted attention due to its usefulness in the establishment of cell death modes and as a biomarker of liver damage rather than as a DAMP molecule.

**Figure 1:**
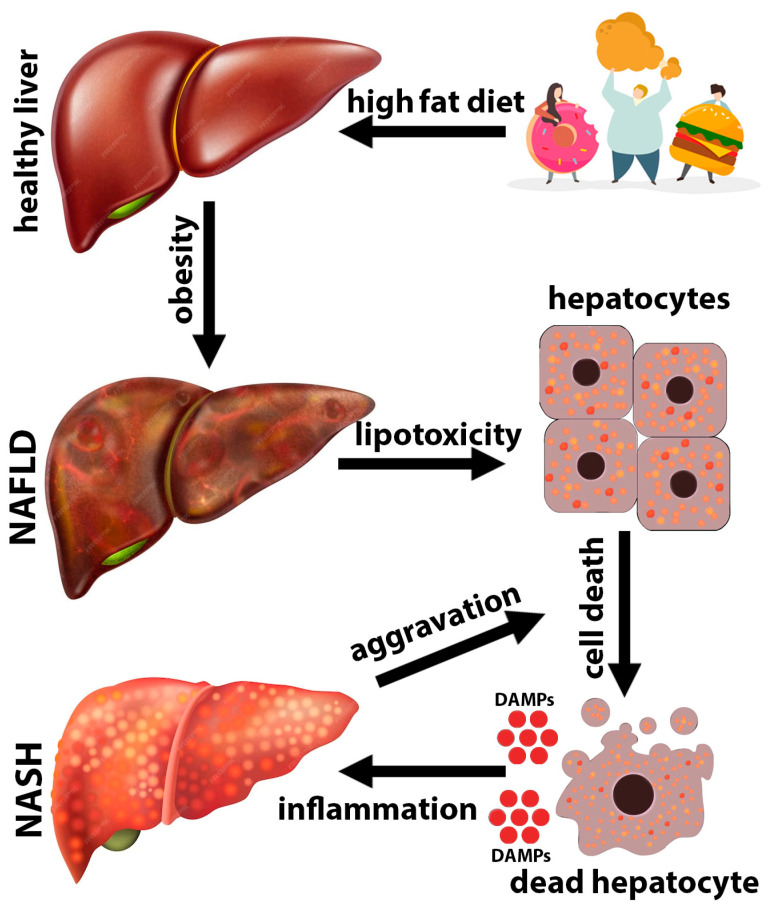
NASH pathogenesis. Excessive consumption of high-fat foods and an unhealthy lifestyle leads to obesity. Obesity is one of the causes of the non-alcoholic fatty liver disease (NAFLD). Excessive accumulation of fats in the liver leads to lipotoxicity and death of hepatocytes. Dead and/or damaged hepatocytes release large amounts of DAMPs, which cause severe inflammation, thereby exacerbating hepatocyte damage.

The high-mobility group protein B1 (HMGB1), which is released from damaged hepatocytes in chronic liver diseases, is probably the most widely assessed DAMP molecule [61]. Many studies have shown that HMGB1 is involved in the pathogenesis of NAFLD. HMGB1 plays a critical role in the initiation and maintenance of a chronic inflammatory state in liver tissue. Since hepatic inflammation is one of the histological features distinguishing simple steatosis and NASH, HMGB1 likely mediates the transition from steatosis to NASH. In NAFLD, lipotoxicity leads to the release of HMGB1 and it is a driver of sterile inflammation [62]. This observation was substantiated using a large cohort study of pediatric patients with biopsy-confirmed NAFLD, which showed higher levels of circulating HMGB1 in children with NAFLD than in obese-only controls. The level of circulating HMGB1 not only correlated with the degree of fibrosis, but also with the levels of inflammatory mediators such as TGF-β and MCP-1 [63]. These results suggest a critical role for HMGB1 in the progression of NAFLD [63]. Therefore, serum HMGB1 may serve as a potential biomarker for the early diagnosis of NAFLD and as a target for therapies aimed at the prevention and treatment of NAFLD-associated inflammation. HMGB1 protein is both a nuclear factor and a secreted protein. In the cell nucleus, it acts as an architectural chromatin-binding factor that bends DNA and promotes protein assembly at specific DNA targets. HMGB1 is secreted by activated monocytes and macrophages and passively released by necrotic or damaged cells [64]. HMGB1 interacts with several receptors such as TLR2, TLR4, and RAGE. TLR4 is the primary receptor for HMGB1 in mediating macrophage activation and cytokine release during tissue injury. Activation of the HMGB1 binding receptors results in a wide range of inflammatory responses, including the production of pro-inflammatory cytokines and the recruitment of immune cells to the site of injury. HMGB1 may also act in conjunction with other pro-inflammatory mediators, such as single-stranded DNA, LPS, IL-1β, and nucleosomes to induce inflammation. Structurally, among the two DNA-binding domains of HMGB1, the B-box recapitulates the inflammatory activity of the full-length protein, whereas the A-box counteracts it [65]. In multiple experimental models of murine liver fibrosis associated with cholestasis, alcoholic steatohepatitis or NASH increased expression and release of HMGB1 are induced [66]. Notably, neutralization of HMGB1 protected against liver fibrosis, whereas injection of recombinant HMGB1 promoted liver fibrosis [66]. Extracellular HMGB1 is capable of activating the RAGE-PI3K-AKT1/2/3 pathway or the RAGE-ERK pathway to upregulate type I collagen synthesis by hepatic stellate cells [66,67]. Importantly, HMGB1 does not stimulate profibrotic signaling through TLR2, TLR4, and TLR9. The receptor specificity in the profibrotic response appears to be distinct from that in liver inflammation, where HMGB1 interacts with RAGE and TLRs. Via the TLR4, HMGB1 secreted from lipotoxic hepatocytes facilitates a paracrine cytolytic effect on neighboring hepatocytes loaded with cholesterol [40,61]. NASH pathogenesis is presented in Figure 1.

Thus, the pathogenesis of NAFLD/NASH starts with the death of hepatocytes and the release of DAMPs from dying cells. DAMPs primarily activate resident hepatic macrophages and, subsequently, together with the cytokines and chemokines released by macrophages, recruit various cells of innate and adaptive immunity to the liver, ultimately leading to massive inflammation.

## 3. Role of Kupffer Cells in the Pathogenesis of NASH

Despite the abundance of data, the pathophysiology of NASH remains incompletely understood at both molecular and cellular level. It is clear, though, that the innate and adaptive immune systems play critical roles in the pathogenesis of NASH. Key players are the pro-inflammatory cytokines such as TNFα, IL-1β, and IL-6, as well as adipokines derived from liver and adipose tissue [68]. These cytokines are the main mediators of hepatic acute phase reaction triggering host intra-liver defenses, but if the problem turns chronic, they become implicated in the development of fibrosis and cirrhosis and participate not only in the deterioration of tissue homeostasis, but also affect liver-specific metabolic functions by altering the gene expression of key regulatory enzymes of the carbohydrate and lipid metabolism [69]. The pro-inflammatory environment in the liver favors the deterioration of very low-density lipoprotein (VLDL) secretion and transcriptional upregulation of de novo lipogenesis, as well as the weakening of mitochondrial fatty acid oxidation, generally increasing lipid accumulation in the liver [70]. It may be surprising that NASH develops in immunodeficient mice that lack B- and T-cells, as well as NK cells. It has become clear that there is a connection between the immune system and the metabolic disorders associated with obesity. The innate immune system senses nutritional stress and mediates the progression of obesity, NASH, and type 2 diabetes. Winkler et al. [71] used an immunodeficient mouse model that allowed the separation of adaptive and innate immune responses and showed that NASH develops even in the absence of adaptive immunity. Since the adaptive immune system cells did not play a significant role in this model, it is likely that the resident liver macrophages and dendritic cells of the innate immune system were the main mediators of inflammatory hepatic steatosis at the cellular level. It is the pro-inflammatory cytokine TNFα, secreted during chronic nutritional overload by the resident liver macrophages, Kupffer cells, that is considered the key player in NASH pathogenesis [72]. Downstream targets of Kupffer cells are hepatocytes and hepatic stellate cells, which are activated and transformed into myofibroblasts in response to TNFα.

Kupffer cells playing the central role in liver tissue inflammation are specialized macrophages lining the walls of the hepatic sinusoids from the lumen side and accounting for about 30% of sinusoidal cells [73]. Kupffer cells carry out five major functions critical for maintaining the liver and whole-body homeostasis. These include (1) clearance of cellular debris and metabolic waste [74,75,76], (2) maintenance of iron homeostasis through phagocytosis of red blood cells and subsequent recycling of iron [77,78,79,80], (3) regulation of cholesterol homeostasis through cholesteryl ester production transfer protein, which is important for reducing circulating levels of high-density lipoprotein-cholesterol and increasing levels of very low-density lipoprotein-cholesterol [81], (4) mediating antimicrobial defense [82,83], and (5) promoting immunological tolerance [84,85].

The origin of Kupffer cells in embryogenesis was described in detail by Li et al. [86]. The Kupffer cell pool formation follows a program that is consistent between mice and humans and occurs in three waves at different stages of embryonic development. Embryonic Kupffer cells reside in the liver throughout life and their population is preserved due to proliferation [87,88]. In adults, the pool of Kupffer cells can be replenished by bone marrow monocytes migrating to the liver and differentiating into Kupffer cells [89].

Kupffer cell populations in mice and humans are heterogeneous. Regrettably, human Kupffer cells do not have uniform markers for identification. In three independent scRNA-seq studies, human Kupffer cells were identified as CD163^+^MARCO^+^CD5L^+^TIMD4^+^ [90,91,92]. Functionally, these cells exhibit anti-inflammatory, anti-tumor, and immunomodulation activity [90,91,92]. Compared to MARCO^-^ macrophages, Kupffer cells express fewer inflammatory (TNFα), but more immunosuppressive genes (e.g., PDL1) when stimulated with LPS or IFN-γ [90]. Another study showed that Kupffer cells display the CD32^int^CD68^+^CD14^+^ phenotype and have the potential to regulate the immune response [93]. In a recent study, VSIG4 was identified as the best marker of human Kupffer cells [94]. Blériot et al. [95] discovered two subpopulations of embryonic Kupffer cells in the mouse liver that differ phenotypically and functionally: a major CD206^lo^ESAM^-^ population (KC1) and a minor CD206^hi^ESAM^+^ population (KC2). KC2 were more likely to express genes involved in metabolic processes in both steady state and diet-induced obesity and hepatic steatosis [95]. Human embryonic Kupffer cells express CD49a, whereas bone marrow derived Kupffer cells do not express this marker. Actually, CD49a can be used to distinguish those two pools of Kupffer cells. Human embryonic Kupffer cells express high levels of both the pro-inflammatory TNFα and IL-12 and the anti-inflammatory IL-10 cytokines, suggesting a dual role in inflammation. However, LPS does not affect the expression of these cytokines in the embryonic Kupffer cells. In contrast, these three cytokines are expressed, though at low levels, in Kupffer cells derived from the bone marrow monocytes and their expression is upregulated by LPS [96]. Therefore, the embryonic Kupffer cells appear to be functional in homeostasis, whereas bone marrow derived Kupffer cells become functional in cases of injury.

In mice, macrophages (MoMϕs) recruited from the bone marrow to the liver have the CD11b^+^F4/80^int^Ly6C^+^CSF1R^+^ phenotype, while the resident Kupffer cells display the CD11b^low^F4/80^high^Clec4F^+^ phenotype [97,98,99,100,101]. MoMϕs differentiate from circulating monocytes, which are derived from bone marrow CX3CR1^+^CD117^+^Lin^−^ progenitor cells [102]. In mouse models of liver disease, hepatic MoMϕs are divided into two major subpopulations according to the level of Ly6C expression: the inflammatory Ly6C^high^ and the restorative Ly6C^low^ MoMϕs. Depending on the signals expressed by the liver microenvironment, recruited MoMϕs can differentiate into cells of different phenotypes. For example, the recruitment of inflammatory Ly6C^high^ MoMϕs specifically relies on the CCL2/CCR2, CCL1/CCR8, and CCL25/CCR9 signaling pathways, with chemoattractants secreted by activated Kupffer cells, hepatic stellate cells, and liver sinusoidal endothelial cells [103,104,105,106,107]. Inhibition or elimination of these signaling pathways in mice results in reduced MoMϕ recruitment, hepatic inflammation, and overall fibrosis [103,108]. However, it should be noted that MoMϕs are highly plastic, as shown by the potential of Ly6C^high^ MoMϕs to switch to the restorative Ly6C^low^ phenotype [109].

**Figure 2:**
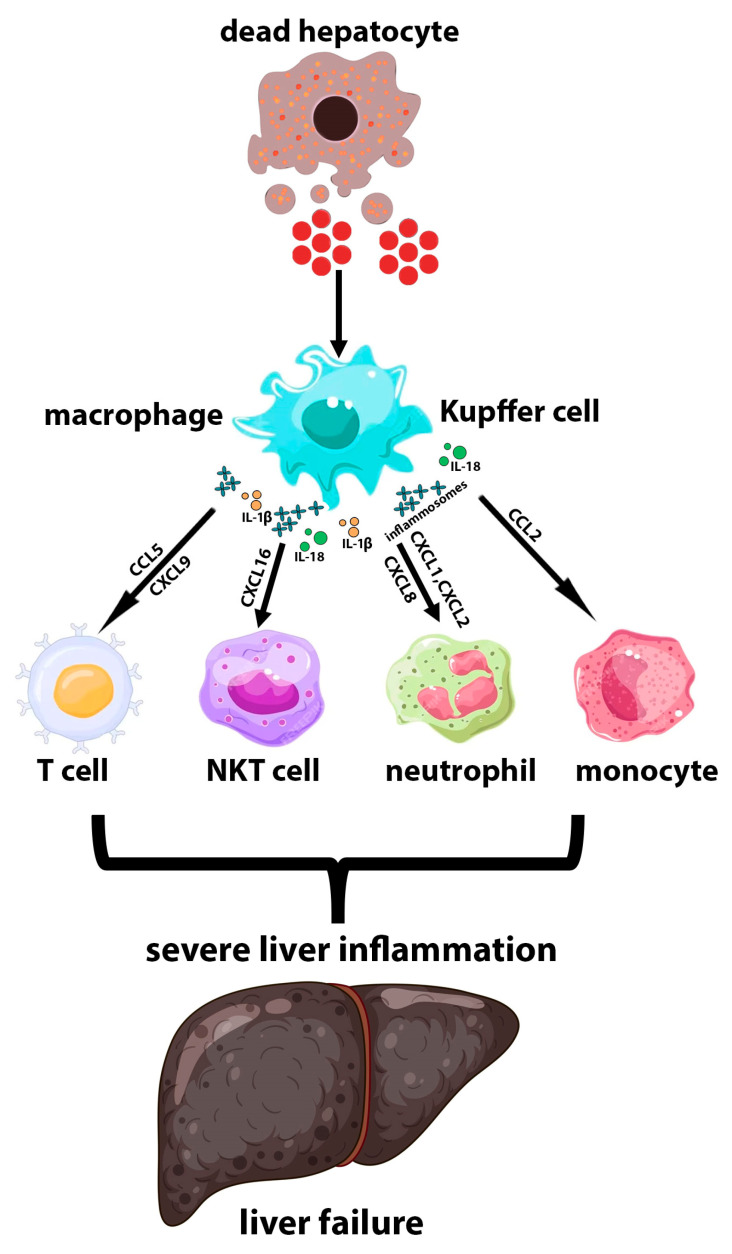
Activation of Kupffer cells in NASH liver. DAMPs released by dead hepatocytes activate Kupffer cells or recruit macrophages, which begin to produce a wide variety of the pro-inflammatory cytokines/chemokines and inflammosomes. Chemokines stimulate the recruitment of the innate and adaptive immune cells to the liver, leading to increased inflammation and further liver damage.

Due to their central position in the hepatic microenvironment, their long cytoplasmic protrusions, and the high density of pattern recognition receptors (PRRs) on their surface, including Toll-like receptors (TLRs) and nucleotide binding oligomerization domain-like receptors (NLRs), Kupffer cells act as the first line of responders for liver damage [110]. Kupffer cells play diverse roles in regulating inflammation in NASH. NASH induces the upregulation of 891 Kupffer cell genes associated with ECM remodeling, lipid metabolism, bacterial clearance, and recruitment of circulating monocytes [111]. Kupffer cells are able to enhance or attenuate hepatic inflammation. Mitochondrial DNA from apoptotic hepatocytes activates the STING/NF-kB signaling pathway in Kupffer cells and leads to the amplification of inflammation [112]. As initial sensors of liver injury, Kupffer cells recruit monocytes, neutrophils, and maybe other leukocytes through the secretion of a variety of chemokines, including CCL2 and CXCL1 [113]. Kupffer cells are the main source of CCL2 [114,115], which attracts CCR2^+^ monocytes to the damaged liver. Kupffer cells also secrete CXCL1, CXCL2, and CXCL8 to attract neutrophils [114], which contribute to hepatic ischemia–reperfusion (IR) injury and heat-induced liver injury [52,113,116]. It appears that infiltrating Ly6C^high^ MoMϕs also release chemokines and promote leukocyte recruitment during liver disease. For example, in mouse models of hepatic fibrosis induced by carbon tetrachloride (CCl_4_) and an MCD diet, Ly6C^high^ MoMϕs express CXCL16 and promote the recruitment of CXCR6^+^ natural killer T (NKT) cells, which exacerbate inflammation and fibrogenesis [117]. In mice fed a high fat diet, Ly6C^high^ MoMϕs produce CCL5 and CXCL9 in a S100 calcium-binding protein A9 (S100A9)-dependent manner. These chemokines lead to liver recruitment of both CD4^+^ and CD8^+^ T cells, which contribute to insulin resistance [118,119]. Studies of chronically inflamed livers from patients with alcoholic liver disease (ALD), NASH, primary biliary cholangitis, or primary sclerosing cholangitis have also shown that intermediate CD14^high^CD16^+^ monocytes (similar to Ly6C^high^ MoMϕs in the murine liver), which are derived from infiltrating classical CD14^high^CD16^−^ monocytes, secrete pro-inflammatory cytokines and chemokines such as TNFα, IL-1β, CCL1, and CCL2 [120]. Figure 2 schematically shows the inflammatory process mediated by Kupffer cells in the NASH liver.

Inflammosomes are multiprotein complexes that can sense danger signals sent by pathogens and damaged cells via TLRs and NLRs. Inflammosome activation induces caspase-1-mediated cleavage and maturation of the IL-1β and IL-18 cytokines [121]. In the liver, gut-derived PAMPs (pathogen-associated molecular patterns), cell damage-induced DAMPs (e.g., ATP), crystals (e.g., cholesterol), palmitic acid, and ROS present the well-characterized signals that induce inflammosome activation in macrophages [35,122,123,124]. DAMPs such as ATP and uric acid, which are released from damaged hepatocytes, induce inflammosome activation in hepatic Kupffer cells in mouse models of ASH and NASH [125,126]. Macrophage inflammosome activation plays an important role in the pathogenesis of chronic liver diseases such as ALD and NASH [127]. IL-1β, which is released as a result of the inflammosome activation in Kupffer cells, plays a critical role in the promotion of alcohol-induced steatosis, inflammation, and liver injury [128]. Deletion of caspase-1 or caspase-1 adapter in mice results in impaired IL-1β production, thereby attenuating ALD symptoms [128]. With regard to NASH, in vitro studies have shown that the NLRP3 inflammosome can sense lipotoxicity-associated increases in intracellular levels of ceramides, cholesterol crystals, saturated fatty acid content, mtDNA levels, and ROS content, causing caspase-1 induction in macrophages and subsequently promoting IL-1β production [129,130,131,132]. In mouse models of NASH induced by supplying various diets, including an atherogenic diet, methionine- and choline-deficient diet (MCD), high fat diet (HFD), and Western diet, activation of NLRP3 inflammosomes and subsequent production of IL-1β exacerbates inflammatory responses while increasing the IL-6 and CCL2 levels and the numbers of infiltrating MoMφ and neutrophils [132]. Genetic deletion or pharmacological inhibition of NLRP3 in mice significantly suppresses tissue inflammation and improves pathological features of NASH such as fibrosis and insulin resistance [129]. Liver samples from patients with NASH showed significantly increased expression of the inflammosomal genes (NLRP3 and caspase-1) compared with normal liver tissue [133] or liver samples from patients with non-alcoholic fatty liver disease [134].

It has been shown that in NASH the pool of embryonic Kupffer cells is reduced with increasing dietary cholesterol [135,136]. A reduction in the number of Kupffer cells is also observed in MCD diet-induced NASH [135] and in hepatocellular carcinoma [137]. This may be caused by apoptosis or cell death induced by the disease [138,139]. During MCD feeding in mice, the pool of the Ly6C^lo^Clec4F^+^Tim4^+^ resident Kupffer cells was significantly depleted, while that of the recruited Ly6C^lo^Clec4F^−^Tim4^−^ monocyte-derived macrophages was enriched. During recovery, the number of resident Kupffer cells normalized, as well as the level of recruited monocyte-derived macrophages returning to the baseline. It is noteworthy that Ly6C^lo^Clec4F^+^Tim4^-^ monocyte-derived Kupffer cells did not self-renew during the recovery period, but were reduced, while recovery from MCD diet feeding was characterized by an increased content of Ki-67^+^ proliferating resident embryonic Kupffer cells [135]. MoMϕs are considered the main contributors to the replenishment of the macrophage pool. This process depends on the concerted actions of hepatic stellate cells and hepatic sinusoidal endothelial cells, which orchestrate monocyte occupancy and the imprinting of the Kupffer cell phenotype, including the expression of the transcription factors ID3 and liver X receptor-alpha (LXR-α) [140,141].

Because hepatic macrophages are the primary component of the immune response during the development of sterile inflammation in the setting of non-alcoholic fatty liver disease, the precise orchestration of their activation, differentiation, and polarization plays a critical role in resolving or triggering subsequent disease progression. As mentioned above, the transition from simple steatosis (mild form of NAFLD) to NASH depends on the development of liver inflammation. Early in the disease, Kupffer cells rapidly divide and secrete cytokines and chemokines such as IL-1, TNF-α, MCP-1, and C-C motif chemokine ligand (CCL)5 [142], reflecting their involvement in controlling the inflammatory response in NASH and their major role in recruiting inflammatory cells to the liver [143,144]. A balance between the states of macrophage polarization is crucial for the development of steatohepatitis. In the reparative phase of acute inflammation, hepatic macrophages can undergo activation into M2 macrophages displaying an immunosuppressive but profibrogenic phenotype. Activated macrophages can produce high levels of TGF-β1 and IL-13, leading to the progression of fibrosis [145]. It has been shown that Kupffer cells can both secrete and respond to the pro-inflammatory cytokines, such as IL-6, and anti-inflammatory cytokines, such as IL-10. The anti-inflammatory M2 macrophages can induce apoptosis of the M1 Kupffer cells, which have been reported to slow down the progression of fatty liver diseases [146]. Additionally, the macrophage-derived IL-10 promoted lipid catabolism can subsequently prevent hepatic inflammation [147]. Macrophages have been implicated in the progression of various stages of NAFLD. For example, a study conducted in a young Korean population demonstrated a high number of CD68 ^+^ Kupffer cells in biopsy specimens from patients with advanced NAFLD [148]. In another study, increased numbers of activated macrophages were found interspersed among injured hepatocytes in children with NAFLD [149]. Kupffer cells can be directly stimulated by the excess of free fatty acids and cholesterol. The chemokines they release (CC-chemokine ligand 1 (CCL1), CCL2, and CCL5) promote the differentiation of monocytes into M1 activated macrophages [150]. In phase II clinical trials, inhibition of CCL2 and CCL5 signaling through selective blockade of CCR2 and CCR5 receptors attenuated the signs of NASH, reduced the levels of circulating biomarkers of systemic inflammation, including high-sensitivity C-reactive protein, IL-6, fibrinogen, and IL-1ß, and counteracted the development of fibrosis [151]. Tacke, in his review article [152], described several effective approaches to targeting hepatic macrophages in the treatment of NASH, including specific inhibitors of inflammatory signaling aimed at suppressing Kupffer cell activation (for example, the ASK-1 inhibitor selonsertib); inhibition of monocyte recruitment through pharmacological strategies to interfere with chemokine signaling, including monoclonal antibodies against chemokines or their receptor(s), receptor antagonists that prevent chemokine binding, inhibition of chemokines by aptamer molecules or small molecule inhibitors blocking chemokine-induced intracellular signaling; and use of novel drug carrier materials that may deliver specific drugs (e.g., gene silencing siRNA, inhibitors of inflammatory signaling, and enhancers of autophagy) to hepatic macrophage subsets in order to inhibit their disease-aggravating phenotype.

Thus, both pools of Kupffer cells and recruited macrophages play a critical role in the pathogenesis of liver diseases. The above-mentioned therapeutic approaches targeting hepatic macrophages, modulating their activity and recruitment, although showing some success, are not completely effective in resolving NAFLD/NASH and other liver diseases. Probably, their less than desired effectiveness is explained by the multifactorial and multidirectional nature of the pathogenetic mechanisms of liver diseases. In this regard, the use of mesenchymal stem cells and their derivatives for therapy might turn out to be a promising approach since the mechanisms of their therapeutic effects are also multifactor and may be influencing multiple pathogenetic links.

## 4. Cell Therapy of NASH Based on the Cross-Talk between Transplanted MSCs and Liver Macrophages

Transplantation of MSCs and MSC derivatives such as exosomes or conditioned medium (CM) is effective in resolving inflammation, oxidative stress, fibrosis, and fatty acid and triglyceride accumulation in various NAFLD/NASH mouse models [153,154,155,156]. This therapeutic action of MSCs and their derivatives can be at least partly attributed to their immunomodulatory effect on hepatic macrophages. Ezquer et al. [157] showed that intravenously administered syngeneic bone marrow derived MSCs prevented the onset of NASH in obese mice. The observed hepatoprotection was not associated with the resolution of metabolic syndrome, but with the prevention of the inflammatory process. Really, the inflammatory cytokines IL-1β, INF-γ, TNF-α, and TGF-β1 mRNA levels were lower in MSC-treated obese mice than in untreated obese mice [157]. Human UC-MSC exosomes intravenously transplanted into mice with MCD-induced NASH improved MCD-induced weight loss and liver damage, and down-regulated pro-inflammatory cytokines TNF-α, IL-6, and IL-1β in the plasma [158]. Compared with control MCD mice, more F4/80^+^ macrophages were recruited to the liver in the UC-MSC exosome group, indicating that exosomes potentially alter the distribution and chemotaxis of macrophages in the injured liver. In addition, the levels of the markers of the anti-inflammatory macrophage phenotype, namely CD206, Arginase-1, and IL-10, were increased in MCD mice compared with control diet mice, and their expression was further enhanced in the exosome-treated group, indicating that in the MCD-induced liver injury external exosomes might increase the number of the anti-inflammatory macrophages or promote macrophage polarization into an anti-inflammatory phenotype [158]. In a murine NASH model induced by supplying a combination of Western diet and the repeated administration of low doses of carbon tetrachloride, intravenous administration of conditioned medium from stem cells derived from human exfoliated deciduous teeth reduced liver fibrosis and inflammation, inhibited hepatocyte apoptosis and activation of inflammatory macrophages, and decreased pro-inflammatory and profibrotic mediators including TNF-α, TGF-β, and CCL-2 [159]. In mice with NASH induced using a Western diet combined with lipopolysaccharide treatment, human adipose tissue-derived MSCs and their sEVs significantly decreased serum ALT levels and inflammatory markers but did not affect fat accumulation in the liver. Also, after administering human MSCs and sEVs an improvement in fibrosis and an increase in anti-inflammatory macrophages were observed in the mouse liver [160]. This mechanism may be related to the ability of MSCs to polarize macrophages into the anti-inflammatory M2 phenotype. The M1 and M2 subsets of macrophages play highly dynamic roles in the progression of NASH [161]. In liver fibrosis, it has been found that M1 macrophages are anti-fibrotic due to their ability to actively phagocytose debris and to degrade connective tissue, while M2 macrophages are profibrotic due to the secretion of the tissue-remodeling factors, including fibronectin-1, coagulation factor XIII, tissue-type plasmin activator, matrix-associated protein betaIG-H3, and insulin-like growth factor (IGF) [162]. In vitro, immortalized E1-MYC 16.3 human ESC-derived MSC-EVs are able to significantly polarize M0 macrophages, promoting their differentiation from human peripheral blood monocytes into M2 (CD68^+^CD206^hi^CD163^hi^ macrophages) but not into M1 (CD68^+^PDL1^hi^CD38^hi^ macrophages). In vivo, these MSC-EVs were shown to induce the enrichment of CD163^+^ M2 macrophages in the livers of high-fat diet (HFD)-induced NASH animals and a decrease in the serum IL-6 levels. However, despite the increase in the number of profibrotic M2 macrophages in the liver and the reduction in plasma IL-6, MSC-EVs did not exacerbate fibrosis, but reduced it, also showing a reduction in the NAFLD activity score (NAS assessed using three parameters: the presence of micro- and macrovesicular fat deposits (steatosis), hepatocellular ballooning, and inflammatory cell infiltration) [163]. However, another study showed that human amnion-derived MSC-EVs reduced the number of CD68^+^ Kupffer cells and CD11c^+^ M1 pro-inflammatory macrophages without affecting the number of CD163^+^ M2 anti-inflammatory macrophages in the livers of rats with HFD-induced NASH. It is likely that amnion-derived MSC-EVs attenuated the inflammatory response in the NASH rat model by suppressing the activation of Kupffer cells, especially M1 macrophages, and down-regulating the expression of the inflammatory cytokines (TNF-α, IL-1β, and IL-6), rather than by shifting the M1/M2 macrophages ratio [164]. In a rat model of sepsis-induced liver injury, the treatment with human umbilical cord-derived MSCs inhibited the activation of Kupffer cells towards M1 phenotype, attenuated TNF-α and IL-6 expression, and promoted IL-4 and IL-10 expression both in vivo in septic rats and in vitro in the LPS-treated Kupffer cells [165].

The MSC-mediated modulation of immune responses at the macrophage level occurs due to paracrine factors secreted by MSCs, such as the anti-inflammatory cytokines IL-10 and TGF-β and a variety of immunosuppressive factors, such as heme oxygenase (HO-1) and iNOS, indoleamine 2,3-dioxygenase (IDO), or prostaglandin E2 (PGE2), and due to the direct cell-to-cell contacts [14]. Wang et al. [166], for example, showed that MSCs can exert a hepatoprotective effect in D-galactosamine-induced liver failure through PGE2-induced regulation of macrophage polarization. PGE2 secreted by mouse bone marrow-derived MSCs inhibited Kupffer cell apoptosis via TLR4-ERK1/2-caspase 3 pathway regulation in mouse non-heart-beating liver transplantation model [167]. Some of the above-named factors secreted by MSCs, including TGF-β and indoleamine 2,3-dioxygenase, induce M2 polarization of macrophages and reduce macrophage-mediated inflammatory responses [168,169]. Mitochondrial transfer from MSCs to macrophages enhances their swallowing ability [170], and also promotes phagocytosis and suppresses the secretion of the pro-inflammatory cytokines [171]. Another mechanism of immunomodulation mediated by MSCs may be related to their ability to suppress mitochondrial fission in Kupffer cells, thereby promoting their M2 polarization. In a hepatic warm ischemia–reperfusion injury (IRI) model, MSCs significantly limited the phenotypic M1 polarization, but enhanced the M2 polarization of Kupffer cells isolated from the ischemic liver, as evidenced by the decreased levels of iNOS and IL-1β transcripts, but increased levels of Mrc-1 and Arg-1 transcripts. MSCs inhibited mitochondrial fission in Kupffer cells, as evidenced by a decrease in the levels of Drp1 and Dnm2. The overexpression of Drp1 in the Kupffer cells promoted mitochondrial fission during IR injury and abolished MSC-regulated Kupffer cell M1/M2 polarization after IR injury, thereby antagonizing the therapeutic effect of MSCs [15]. Table 1 presents data on the effects exerted by MSCs or MSC derivatives on macrophages/Kupffer cells.

In the murine traumatic hemorrhagic shock model, MSC-derived extracellular vesicles containing IL-10 mainly accumulated in the liver, where they were captured by Kupffer cells and induced the expression of the protein tyrosine phosphatase non receptor 22 (PTPN22) [172], which had been shown to negatively regulate the pro-inflammatory macrophage activation [173]. It subsequently shifted Kupffer cells to the anti-inflammatory phenotype and mitigated liver inflammation and injury [172]. It is known that after transplantation MSCs quickly die within a few hours [174]. A number of studies have shown that those dead MSCs are engulfed by macrophages with the non-classical Ly6C^low^ phenotype found in the lungs and liver. In vitro experiments showed that following the phagocytosis of UC-MSCs classical human CD14^++^/CD16^−^ monocytes polarized into the non-classical CD14^++^CD16^+^CD206^+^ monocytes. Such non-classical or M2 macrophages began to express the PDL-1 and IL-10, while the expression of the pro-inflammatory TNF-α was reduced [175]. That is, phagocytosed MSCs induce the M2 polarization of macrophages. Direct cell-to-cell contacts between MSCs and macrophages due to the interaction of CD47 present on the MSCs and SIRPα displayed by the macrophages led to a reduction in the liver tissue inflammation through the inhibition of inflammosomes in Kupffer cells in vivo in the liver IRI model and in vitro in the LPS-treated macrophages. The knockout of CD47 in MSCs increased the expression of NEK7, NLRP3, ASC, and cleaved caspase-1 in Kupffer cells in damaged liver and in macrophages in vitro, and also increased pro-inflammatory cytokines and chemokines, including IL-1β, TNF-α, IL-6, CXCL2, and CXCL10 in macrophages after coculture [176].

**Table 1:** The effects of MSCs/MSC derivatives on macrophages/Kupffer cells.

MSCs/MSC Derivatives	Effect on Kupffer Cells/Macrophages	References
PGE2	Inhibition of apoptosis via TLR4-ERK1/2-caspase 3 pathway regulation	[167]
TGF-β	Induction of M2 polarization	[168]
Indoleamine 2,3-dioxygenase	Induction of M2 polarization	[169]
Mitochondria	Enhancement of swallowing ability	[170]
	Phagocytosis promotion and pro-inflammatory cytokines secretion suppression	[171]
MSCs	Inhibition of Drp-1 dependent mitochondrial fission and promotion of M2 polarization	[15]
MSC-derived extracellular vesicles containing IL-10	Induction of the expression of the protein tyrosine phosphatase non receptor 22 (PTPN22) and inhibition of the pro-inflammatory macrophage activation	[172]
Dead MSCs	Induction of M2 polarization	[175]
Direct cell-to-cell contacts via CD47 on MSCs and SIRPα on Kupffer cells	Inhibition of inflammosomes in Kupffer cells	[176]

According to the clinicaltrials.gov website, only one study using mesenchymal stem cells to treat patients with NASH has been enrolled and completed to date (NCT03963921). The complete results of this study have not yet been published. But brief results of this study presented at the EASL Congress 2023 in Vienna showed that the highest dose of human allogeneic liver-derived progenitor cells (HepaStem^®^) were safe and well tolerated by adult patients with either F3 or F4 NASH (SAF fibrosis score). A preliminary efficacy study demonstrated normalization of ALT and AST serum levels and gradual decreasing of bilirubin and triglyceride levels as well as IL-6 levels and fibrosis over a period of 6 months [177]. In the early- and late-stage NASH mouse models, these human allogeneic liver-derived progenitor cells exhibited pronounced anti-inflammatory and anti-fibrotic effects [178]. In another clinical trial (UMIN000022601), MSCs showed a significant therapeutic effect in patients with NAFLD/NASH. After the autologous adipose tissue-derived regenerative (stem) cells’ (ADRCs) transplantation for the treatment of liver cirrhosis due to non-alcoholic steatohepatitis or fatty liver disease, six out of seven patients showed an improved serum albumin concentration and five out of seven patients showed improved prothrombin activity. In one patient, hepatocyte steatosis decreased by one point, and in another single patient, the lobular inflammation index decreased by one point [179].

## 5. Conclusions

Data reviewed in this article can be summarized in the following way. It has been unequivocally demonstrated that NASH entails massive hepatocyte death, which is the prime cause and driver of the subsequent pathological inflammation and fibrosis of liver tissue. Decaying hepatocytes release a variety of biologically active substances, including those constituting the so-called damage-associated molecular patterns (DAMPs) capable of activating different types of cells, including immune cells, most importantly resident liver macrophages (Kupffer cells), as well as macrophages recruited from the blood flow. Activated macrophages produce and release a wide spectrum of cytokines and chemokines, some of which are the attractants of neutrophils and cells of the adaptive immune system, while the others intensify inflammation and promote fibrosis. These events drastically exacerbate disease progression. Therefore, Kupffer cells and recruited macrophages present in the liver tissue constitute the first line of the immune response in NASH and trigger all the following pathological developments. If so, they can be regarded as a prospective target for the development of new medicines and therapeutic approaches.

The progression of NAFLD/NASH is strongly dependent on the numerical ratio of the pro-inflammatory (M1) and the anti-inflammatory (M2) macrophages present in the liver tissue and, accordingly, on the cytokines and chemokines they produce. MSCs or MSC derivatives shift the balance towards the anti-inflammatory M2 phenotype both in vitro and in vivo, substantially reducing liver inflammation in the latter case. MSCs/MSC derivatives affect the liver tissue in multiple ways and even if some of the effects are potentially harmful, they can be neutralized. Thus, the M2 macrophages are profibrinogenic, but with MSC therapy fibrosis is offset due to the parallel inhibition of the stellate cell and pro-inflammatory immune cell activation and the hepatocyte protection against apoptosis. The multifactorial and multidirectional nature of the MSCs/MSC derivatives-based NASH therapy is its main advantage over the other Kupffer cell-targeted approaches under development.

Accumulating preclinical and clinical data regarding the pathogenesis of NASH and the treatments currently under development indicates that the pathogenesis of this disease is complicated and, therefore, effective therapeutic approaches probably need to be designed to simultaneously achieve several goals. Targeting a single link within the NASH pathogenetic complex, as was done in the pan-caspase inhibitor trials, not only does not produce the desired therapeutic effect, but may lead to enhanced disease progression. Therefore, multi-target cell therapy using MSCs and MSC derivatives may prove to be effective.
